# Competition among native and invasive *Impatiens* species: the roles of environmental factors, population density and life stage

**DOI:** 10.1093/aobpla/plv033

**Published:** 2015-04-01

**Authors:** Jan Čuda, Hana Skálová, Zdeněk Janovský, Petr Pyšek

**Affiliations:** 1 Department of Invasion Ecology, Institute of Botany, The Czech Academy of Sciences, Průhonice CZ-252 43, Czech Republic; 2 Department of Ecology, Faculty of Science, Charles University in Prague, Viničná 7, Prague CZ-128 44, Czech Republic; 3 Department of Botany, Faculty of Science, Charles University in Prague, Benátská 2, Prague CZ-128 01, Czech Republic

**Keywords:** Alien species, balsam, competition, congeners, plant density, shading levels, water availability

## Abstract

Many invasive species are considered competitively superior to native species, with the strongest competition expected in species with similar niches and/or in closely related species. However, competition outcome is strongly context-dependent as competitive strength varies along environmental gradients, and life stages, and also depends on abundances. To explore the importance of these factors, we examined competition effects in an experiment with three *Impatiens* species (Balsaminaceae) widespread in central Europe and sharing similar life-history characteristics and habitats: the native *I. noli-tangere*, and two invasive species, *I. parviflora* and *I. glandulifera*. We compared their competitive strength and reciprocal impacts under two levels of water and light availability, two overall planting densities and three competitor densities. We assessed species performance (ability to complete the life-cycle, biomass and fecundity) and temporal competition dynamics in a garden pot experiment. Environmental variables had lower explanatory power than overall planting and competitor density, which indicates the importance of competitive interactions when evaluating plant performance and potential invasion success. Despite poor and delayed germination, the invasive *I. glandulifera* attained dominance even at a high competitor density and was competitively superior across all treatments, exceeding the height of both congeners. *Impatiens parviflora* was competitively weakest, having a negligible impact on both native *I. noli-tangere* and invasive *I. glandulifera*. The intermediate competitive strength of the native *I. noli-tangere* probably results from its intermediate height, and good germination rate and timing. The difference in height among species increased during the experiment when *I. glandulifera* was involved; this species continues growing until autumn, enhancing its competitive superiority. The results provide a mechanistic understanding for the competitive exclusion of native *I. noli-tangere* that occurs in stands with *I. glandulifera*, but the limited impact of *I. parviflora* on *I. noli-tangere* in their mixed stands.

## Abstract (precis)

We examined competition effects in an experiment with three *Impatiens* species (Balsaminaceae) sharing similar life-history characteristics and habitats: the native *I. noli-tangere*, and two invasive species, *I. parviflora* and *I. glandulifera*. The results suggest that the effect of competition on the performance of invasive *Impatiens* species exceeds that of environmental factors, i.e. light and soil moisture. Competitive interactions with co-occurring congeners may thus be a more important predictor of the invasion success of an invasive species and its population dynamics than its response to abiotic factors, and should be taken into account when evaluating their invasion potential.

## Introduction

Non-native species have to overcome numerous barriers to naturalize and become invasive in the introduced range (Richardson *et al*. 2000; Blackburn *et al*. 2011). While immediately after introduction into a new range the species need to cope with the local environment, especially climatic conditions (Wiens and Graham 2005), later on different mechanisms involving interactions, or their absence, with resident biota come into play. It has been suggested that some invading species can exploit resources not used by plants in resident communities (empty niche hypothesis; Elton 1958; Lambdon *et al*. 2008), which results in minimizing or even avoiding competitive interactions with co-occurring species (MacArthur 1972; Crawley 1987; MacDougall *et al*. 2009). On the other hand, the outcome of interactions with resident organisms, especially competition (Levine *et al*. 2004), have been repeatedly found to be important for successful invasion of local communities (Sakai *et al*. 2001; Levine *et al*. 2004; Vilà and Weiner 2004; Maherali and Klironomos 2007; Hierro *et al.* 2011). In particular case studies, competitive advantage of invading species is often attributed to traits such as high germination rate, good survival, fast growth, early or late flowering, high fecundity and tall stature (Baker 1965; Pyšek and Richardson 2007; Kubešová *et al*. 2010; Moravcová *et al*. 2010; van Kleunen *et al*. 2010; Pellock *et al*. 2013).

Nevertheless, alien invaders were not found to be significantly competitively superior to native species in an analysis of available case studies (Daehler 2003). The outcome of competition depended on the environmental context (Daehler 2003), e.g. on water availability (Franzese and Ghermandi 2014), shading (Molina-Montenegro *et al*. 2012) or nutrient supply (Powell and Knight 2009). The competitive hierarchy of alien and native species changes along environmental gradients (Milberg *et al*. 1999; Shea and Chesson 2002; Pathikonda *et al*. 2009), with competitive strength of invaders usually decreasing towards more extreme conditions, such as, for example, high altitudes (Daehler 2005; Alexander *et al*. 2011; Pyšek *et al*. 2011). However, many invasive species possess a high phenotypic plasticity which makes them capable of adapting to a wide range of environmental conditions (Funk 2008; Berg and Ellers 2010). This corresponds to invasive plants often being generalists with a broad tolerance of ecological conditions, but exploiting resources less effectively than specialists (Richards *et al*. 2006).

The strength of competition between species depends on the degree to which their niches overlap (Hutchinson 1957), with two species occupying the same niche unable to co-exist over the long term (Hardin 1960). The strongest competition is expected in closely related species (Darwin 1859; Elton 1946; Maherali and Klironomos 2007; Violle *et al*. 2011). Nevertheless, some studies have found no linkage between the relatedness of competing species and the competition strength (Cahill *et al*. 2008) or reported even an opposite pattern, with less intense competition between closely related species (Diez *et al*. 2008; Mayfield and Levine 2010). Strong competition was suggested as the reason why invasive species from families with numerous members in native floras are under-represented in floras of target regions (Rejmánek 1996; Daehler 2001).

Despite some studies testing for the competitive superiority of invasive plants over native plants under a range of environmental conditions (Powell and Knight 2009; Molina-Montenegro *et al*. 2012; Franzese and Ghermandi 2014), the competitive relationships between these two groups of species have rarely been tested along the gradient of competitor densities (but see Leger and Espeland 2010). Moreover, studies focusing on a reciprocal impact of native species on invasive species are still the exception rather than the rule (Leger and Espeland 2010; Carvallo *et al*. 2013).

The rationale of our study stems from the well-established notion that high-density results in severe competition for resources (Antonovics and Levin 1980; Silvertown and Charlesworth 2009). Invasive species often gain an advantage over their native competitors under high resource supply, but stressful conditions can reverse the hierarchy, leading to a competitive advantage of natives (Daehler 2003). Density-dependent effects may differ across life stages, with the strongest effect found in the emergence stage (Goldberg *et al.* 2001). For intraspecific competition, high plant density usually decreases biomass and the number of individuals (Antonovics and Levin 1980). On the other hand, density effects have rarely been found to be significant in interspecific competition (Connolly *et al*. 1990, but see Antonovics and Fowler 1985).

To obtain a deeper insight into competitive interactions between native and invasive species under manipulated environmental conditions, varying plant densities and different life stages, we used three annual *Impatiens* species occurring in Central Europe: native *I. noli-tangere* and invasive *I. glandulifera* and *I. parviflora*. Using congeners minimizes phylogenetic biases (Burns 2004; Grotkopp and Rejmánek 2007; van Kleunen *et al*. 2010) as well as those associated with other traits such as life history or dispersal mode. Due to the overlap of the species' niches, which brings them into direct contact in the field (Čuda *et al*. 2014), we expected strong interspecific competition to occur (Matesanz *et al.* 2011). It is of interest to understand the ecological interactions among the *Impatiens* species because highly invasive *I. glandulifera*, which has historically colonized river banks, is currently spreading into novel habits such as clearings and roadsides distant from the river courses (Hejda and Pyšek 2006; Čuda *et al*. 2014). Since environmental conditions and dispersal vectors in these novel habitats differ from those acting in river corridors, the competitive interactions among this invader and co-occurring species might be changing. It is thus necessary to establish the competitive hierarchies of the three species across a range of seed availability and environmental conditions. A previous study (Čuda *et al*. 2014) revealed that shade, and moisture drive *Impatiens* distributions in the field. As such, we assessed these factors in a common garden experiment designed to capture the reciprocal effects of competition between species. Specifically, we answer the following questions. (i) What is the effect of density-dependent congeneric competition and environmental conditions on the ability of plants to complete their life-cycle? (ii) How do these factors affect plant biomass and fecundity? (iii) How does the effect of competition change over time with respect to the life stages?

## Methods

### Studied species

All three studied *Impatiens* (Balsaminaceae) species are annuals with similar biological characteristics (Coombe 1956; Beerling and Perrins 1993; Hatcher 2003) and habitat preferences (Slavík 1997), but with different origin and invasion status in the Czech Republic (Pyšek *et al*. 2012*a*). They partly differ in germination rates and stratification demands (Perglová *et al*. 2009), but in the field the majority of seedlings emerge within one month (April) (J. Čuda, pers. obs). The presence of all three species is dependent on disturbances and they therefore often occur in early successional herbaceous communities. At the same locality, the spatial pattern of the occurrence of individual *Impatiens* species is driven by canopy closure and water availability (Čuda *et al*. 2014).

*Impatiens noli-tangere* L., a native species, grows in damp forests, at clearings, along watercourses and around springs (Slavík 1997). It is recorded from 39 habitat types in the Czech Republic (Sádlo *et al*. 2007). Its height varies depending on local conditions from 20 to 120 cm (Hatcher 2003). The plants flower from July to August and set seed from mid-July to end of August. It is reported that it may be suppressed by competition from invasive *I. parviflora*, with which it often co-occurs (Tichý 1997; Faliński 1998; Chmura and Sierka 2007) as well as by competition from *I. glandulifera* (Vervoort *et al*. 2011; Čuda *et al*. 2014).

*Impatiens parviflora* DC., an invasive species, is characterized by a height similar to that of *I. noli-tangere* (Coombe 1956) and a broad ecological amplitude, being recorded from 45 habitat types in the Czech Republic (Sádlo *et al*. 2007; Pyšek *et al*. 2012*b*). It often grows as a dominant in nitrophilous herbaceous vegetation at shady mesic sites, in alluvial forests, oak-hornbeam forests, ravine forests and spruce or *Robinia pseudoacacia* plantations (Pyšek *et al*. 2012*b*). The plants flower from mid-June to October, setting seed from late June until the first autumn frosts.

*Impatiens glandulifera* Royle, a highly invasive species, occurs predominantly along rivers, but has been recently colonizing forest clearings and margins, wet ditches, forest roads and ruderal sites. It is recorded from 16 habitat types (Sádlo *et al*. 2007; Pyšek *et al*. 2012*b*; Pahl *et al*. 2013) but the number is expected to increase due to the ongoing spread. The plants flower from late July until the first frosts, setting seeds from late August. Due to high seed production (Moravcová *et al*. 2010) and tall stature up to 3 m (Adamowski 2008), it is highly competitive and able to replace the native flora in invaded sites.

### Seed collection

Seeds were collected from large established populations, extending over 2500 m^2^ in July and August 2011. Seeds of *I. glandulifera* were collected in Bohuslavice nad Metují (50°18′4.315″N, 16°5′22.730″E) along a riverbank and a meadow margin partly shaded by trees; *I. parviflora* and *I. noli-tangere* in Velký Osek (50°6′42.770″N, 15°10′10.635″E) in a flooded forest and forest gaps. Due to the low seed production, seeds of *I. noli-tangere* were collected also in Peklo by Nové Město nad Metují (50°21′28.501″N, 16°9′48.858″E) in a flooded forest and clearings and mixed together with those from Velký Osek. Altogether at least 15 000 seeds from at least 1000 individuals of each species were taken. After the collection, seeds of *I. noli-tangere* were kept in refrigerator at 3 °C on heat-sterilized wet river sand in the Petri-dishes as dry storage decreases the seed germination considerably (Perglová *et al*. 2009). Seeds of *I. parviflora* and *I. glandulifera* were stored in paper bags at room temperature.

### Experimental design

The experiment was carried out in the experimental garden of the Institute of Botany ASCR in Průhonice (49°59′38.972″N, 14°33′57.637″E), 320 m above sea level, temperate climate zone, where the mean annual temperature is 8.6 °C and the mean annual precipitation is 610 mm. The seeds of the three *Impatiens* species were sown, separately or in pairs, into 20 × 20 × 23 cm^3^ pots with ∼5 L of heat-sterilized common garden soil in early November 2011. Sowing seeds in the autumn ensured cold stratification, required for breaking the dormancy of the seeds (Perglová *et al*. 2009). Seeds were homogeneously dispersed on the soil surface and covered with a thin layer (0.5 cm) of soil. Seeds were sown to achieve two different total densities of seedlings that correspond to the range of densities typically observed in the field (J. Čuda, unpubl. data): high density (60 seedlings per pot) and low density (12 seedlings per pot). Within each density level, we sowed seeds to create three ratios of target plants to competitor plants, such that target plants experienced high (1 : 5), medium (1 : 1) and low (5 : 1) levels of competition from their congeners. Including also no-competitor (monospecific) controls resulted in 24 species-density-competition combinations (see Table 1).

**Table 1.** Seed doses of target species and competitor under different levels of total plant and competitor density. *The number of seeds was increased in species where we expected poor germination (Perglová *et al*. 2009) to achieve comparable numbers of emerged seedlings. In *I. noli-tangere* the number of seeds was enhanced from two to four and from six to eight, and in *I. parviflora* from two to three and from six to seven.

Total density	Competitor density	Number of seeds of target species	Number of seeds of competitor	Final ratio (target : competitor)
High	High	10	50	1 : 5
	Medium	30	30	1 : 1
	Low	50	10	5 : 1
	No competitor	60	0	1 : 0
Low	High	2*	10	1 : 5
	Medium	6*	6*	1 : 1
	Low	10	2*	5 : 1
	No competitor	12	0	1 : 0

In order to test the influence of environmental factors on species performance and competitive interactions, plants were grown under two water and shading levels in a full factorial design, hereafter referred to as moderate shade/low water; deep shade/low water; moderate shade/high water and deep shade/high water treatments. Due to logistic reasons plants exposed to the same treatment were grown together in the same experimental bed. The experimental design therefore consisted of a total of four experimental beds. Plants under high water treatment were watered twice a day in the morning and evening with tap water. The low water treatments were watered only when plant wilting was noticed. The aim was to induce water stress in the low water treatment and to provide full water supply in the high water treatment. The average soil moisture was 21.2 % in the low water treatments and 29.6 % in the high water treatments. The moisture was measured only once in every fifth pot (to obtain information about the difference between the treatments, not for the purpose of an analysis) on 20 June 2012, one day after the last rain and ∼6 h after watering the plants in the morning. Shading levels were achieved by using a green garden shading net transmitting 10 and 65 % of incident radiation, without any significant change in light spectrum, for deep and moderate shade, respectively.

In total, the experiment consisted of 960 pots (4 environmental treatments × 24 species-density-competition combinations × 10 replicates). In all four beds, pots containing *I. glandulifera* plants (both no-competitor controls and pairs) were placed in separate sections, separated by 1 m from pots without it, to avoid unwanted shading by tall *I. glandulifera*. Pots were randomized within the sections and separated by 20 cm.

Unfortunately, very low emergence of *I. glandulifera* seedlings was recorded in the deep shade/high water bed. This was probably due to an anomalous warm episode in January when some of the seeds of *I. glandulifera* germinated and were killed afterwards by frost. The frost affected only this one bed probably because it was located lower on the slope than the others and could be exposed to cooler air accumulating in the lower part of the garden. Thus, we excluded the bed from all analyses.

The first sampling was carried out on 3–4 April 2012, after the seedlings emerged in the majority of pots, and the number of plants was recorded. Later samplings were done in 3-week intervals: April 26–27, May 14–16, June 4–5, and the number of plants and their mean height (taken as the height of the layer with the maximum density of leaves) were recorded. Plants were harvested in July, when they reached maximum size and the first symptoms of senescence appeared in *I. noli-tangere* and *I. parviflora*: after recording the same characteristics as on previous samplings, the plants were clipped at soil level and sorted by species. As capsules are released after seed maturation and only peduncles remain attached to the stem, we used peduncles as a proxy of the reproductive output. The peduncles were clipped from the plants, and counted. For technical reasons (extreme time demand), peduncles were analysed only in 60 % of the sections in each of moderate shade/low water and deep shade/low water treatments. The complete biomass, i.e. that of vegetative parts and peduncles, was dried at 70 °C for 24 h and weighed.

### Datasets and statistical analyses

We arranged the data collected during the experiment into three datasets (Table 2).The first one, hereafter ‘vegetative dataset’, was used to analyse the effects of experimental conditions on ‘life-cycle completion’ (number of individuals per species in the pot at the time of harvest divided by the number of sown seeds) and on the average biomass of the individual (further referred to as ‘biomass’). Because almost all surviving individuals were fruiting at the time of the harvest, we took the number of surviving individuals as equal to the ability to complete the life-cycle. The second one, hereafter ‘reproductive dataset’, focused on the effects of experimental conditions on the average number of capsules produced by an individual (further referred to as ‘fecundity’). The third one, hereafter ‘temporal dataset’ was used to explore changes in plant height under competition for light among the *Impatiens* species over the duration of the experiment. The response variable was the height ratio of the target species (*t*) to the competitor (*c*) and target species and calculated as *t*/(*c* + *t*). Unlike the simple ratio target species/competitor species known to have the Cauchy distribution, this response variable comes from a β distribution, which can be approximated by normal distribution (and thus linear regression can be used; Sokal and Rohlf 1987). All *Impatiens* species were tested separately in all analyses.

**Table 2.** Overview of analyses within the study. Life-cycle completion = number of individuals per species in the pot at the time of harvest divided by the number of sown seeds; biomass = mean weight of individual at the time of harvest; fecundity = mean number of capsules per individual at the time of harvest; temporal variation = mean height of individuals of target species divided by mean height of individual of competitor at the four time-sequential measurements. ^1^Shade and water levels.

Analysis	Response variables	Explanatory variables	Data
(1) Life-cycle completion	Number of individuals in the time of harvest/number of seeds	Density, environmental treatment^1^, competitor identity, competitor density	Vegetative dataset
(2) Biomass	Mean weight of individual	Density, environmental treatment^1^, competitor identity, competitor density	Vegetative dataset
(3) Fecundity	Mean number of capsules per individual	Density, shading treatment, competitor identity, competitor density	Reproductive dataset
(4) Temporal variation	Target species height/(target species + competitor height)	Pot (covariable), time, density, environmental treatment^1^, competitor identity, competitor density	Temporal dataset

All three datasets were analysed by means of linear regressions. The competitor density was expressed as the number of emerged competing individuals in the pot and used as a continuous variable in the analyses. The effects of environmental treatment and competitor identity were further tested by Tukey HSD post-hoc comparisons. Some of our response variables were ratios (life-cycle completion and temporal variation), where the underlying statistical distribution generating the data is binomial or β, respectively, however, the observed values lay within the range of 0.2–0.8, where linear approximation of functional relationships and assumption of normal distribution of errors is relatively reasonable (Crawley 2007). The assumptions of linear regression were checked by plotting the diagnostic graphs (Crawley 2007). All response variables with the exception of the life-cycle completion and temporal variation analyses had to be log-transformed in order to meet the assumption of homogeneity of variance. The estimates of life-cycle completion differed in their precision among the pots, since they were based on different numbers of seed sown or capsules produced (respectively). This was reflected in the analysis by setting these totals as weights in the corresponding linear regressions. We included the pot identity in the analysis of the temporal dataset in order to account for hierarchical structure in data (i.e. four repeated measurements from an individual pot, see Table 2). Given the pseudoreplication of our environmental treatments, responses to environmental conditions should be interpreted with caution. All computations were undertaken in the R 2.15.3 statistical environment (R Core development team, available at www.r-project.org).

As some response variables (biomass and fecundity) were calculated as the mean value per individual, they were strongly influenced by total density according to the law of constant final yield (Harper 1977). Because individuals from the low-density treatment are bigger and more fecund, we focused on the effects of competition density and environment (Figs 2A–C and 3A and B). In the results (Table 3), we present only the influence of the three strongest factors (explaining the majority of variance) to each response variable **[see Supporting Information for details]**.

**Table 3.** The influence of three strongest factors from analysis of particular species (according to explanatory power) on *Impatiens* fitness. D.f., residual degrees of freedom; E.V. total, variability explained by the model. For effects direction and explained variability by the particular factor **[see Supporting Information—Tables S1–S4]**. For the explanation of response variables see Table 2; factors are described in Methods.

	Life-cycle completion	Biomass	Fecundity	Temporal variation
*I. noli-tangere*				
Factor 1	Environment	Density	Density	Time × competitor identity
Factor 2	Density × environment	Competitor identity	Competitor identity	Time × density
Factor 3	Environment × competitor identity	Environment	Shading	Time × competitor density
D.f.	371	351	139	862
EV total (%)	35.5	54.7	55.0	58.7
*I. parviflora*				
Factor 1	Environment	Competitor density	Competitor identity	Competitor identity
Factor 2	Competitor identity	Density	Density	Time
Factor 3	Environment × competitor density	Competitor identity	Competitor density	Time × competitor density
D.f.	346	341	139	772
EV total (%)	27.7	58.8	50.2	49.4
*I. glandulifera*				
Factor 1	Environment × competitor identity	Density	–	Time
Factor 2	Competitor identity	Competitor identity	–	Time × competitor identity × environment
Factor 3	Density × competitor identity	Environment	–	Time × environment
D.f.	284	277	115	689
EV total (%)	23.9	22.7	14.9	70.7

## Results

### Effect of competition and environment on life-cycle completion

**Figure 1.**
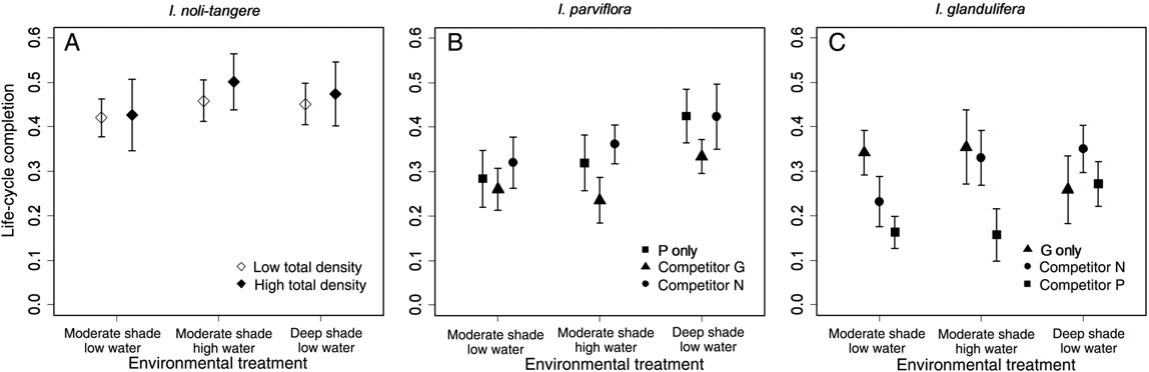
(A–C) Effect of competition (competitor identity and competitor density), total plant density (low and high) and environmental conditions (water and shading) on life-cycle completion rate (number of individuals per species in the pot at the time of harvest divided by the number of sown seeds). Symbols show species mean value under interspecific competition or without it; error bars show the 95 % confidence intervals. Species abbreviations: N = *I. noli-tangere*, P = *I. parviflora*, G = *I. glandulifera.* Each graph shows the pair of most important variables (according to the explanatory power). Sixty seeds were sown into pots with high total plant density and 12 into pots with low total density (A).

Life-cycle completion was affected more strongly by environment than by competition (Table 3) **[see Supporting Information—Table S1]**. The highest proportion of *I. noli-tangere* individuals completed their life-cycle under high water and high total density (Fig. 1A). *Impatiens parviflora* performed better in deep shade (Fig. 1B), but poorly in competition with *I. glandulifera*, with the negative effect of the latter species being significant in all environments. The lowest number of *I. glandulifera* individuals completed their life-cycle in competition with *I. parviflora*, but only in moderate shade treatments (Fig. 1C).

### Effect of competition and environment on biomass and fecundity

**Figure 2.**
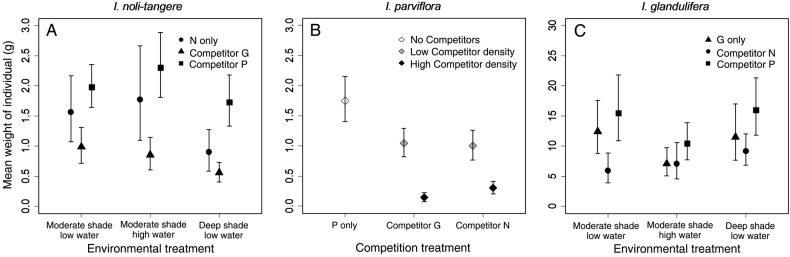
(A–C) Effect of competition (competitor identity and competitor density) and environmental conditions (water and shading) on biomass. Symbols show species mean value under interspecific competition or without it; error bars show the 95 % confidence intervals. Species abbreviations: N = *I. noli-tangere*, P = *I. parviflora*, G = *I. glandulifera*. Each graph shows the pair of most important variables (according to their explanatory power). To visualize the effect of competitor density (B), we divided this continuous variable into two categories: low competitor density = under mean competitor number and high competitor density = above mean competitor number.

Unlike life-cycle completion, biomass and fecundity was affected more strongly by competition than by environment (Table 3) **[see Supporting Information—Tables S2 and S3]**. Biomass per individual of all three species was higher in low than high total density treatments **[see Supporting Information—Table S2]**. *Impatiens glandulifera* had considerably higher biomass than the other species and was the strongest competitor, in terms of reducing the other species’ biomass (Fig. 2A–C). Competition with *I. parviflora* increased the biomass of *I. noli-tangere* in all environments relative to the control (Fig. 2A). *Impatiens parviflora* was the weakest competitor with its biomass reduced by both competitors. This decrease was proportional to the competitor density (Fig. 2B). The biomass of *I. glandulifera* increased by competition with *I. parviflora* and was reduced by competition from *I. noli-tangere*. In the high water treatment, the biomass of *I. glandulifera* was low irrespective of competitors (Fig. 2C).

**Figure 3.**
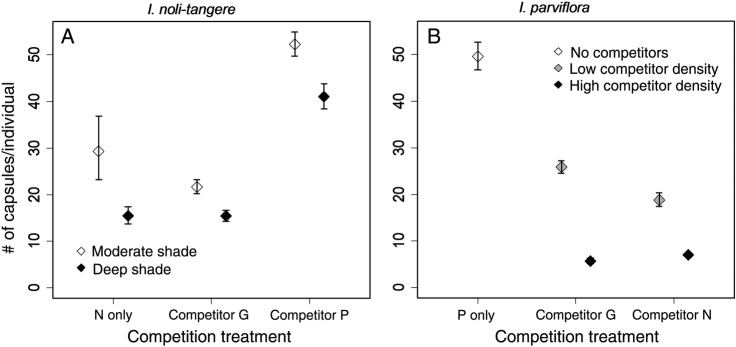
(A, B) Effect of competition (competitor identity and competitor density) and environmental conditions (shading) on fecundity (number of capsules per individual). Symbols show species mean value under competition; error bars show the 95 % confidence intervals. Each graph shows the pair of most important variables (according to their explanatory power). To visualize the effect of competitor density (B), we divided this continuous variable into two categories: low competitor density = under mean competitor number and high competitor density = above mean competitor number. We omitted the graph for *I. glandulifera* for which no significant effects of competition were found.

Fecundity, i.e. the number of capsules per individual, was higher in *I. noli-tangere* and *I. parviflora* under lower densities **[see Supporting Information—Table S3]**. The fecundity of *I. noli-tangere* was higher in competition with *I. parviflora* and lower in that with *I. glandulifera* than without competitors in addition, *I. noli-tangere* plants were more fecund under moderate than deep shade (Fig. 3A). *Impatiens parviflora* was less fecund if the density of competitors was high; both congeners had such negative effects (Fig. 3B). None of the tested factors affected the fecundity of *I. glandulifera* (Table 3) **[see Supporting Information—Table S3]**.

### Temporal variation in competition due to the differences in species height

**Figure 4.**
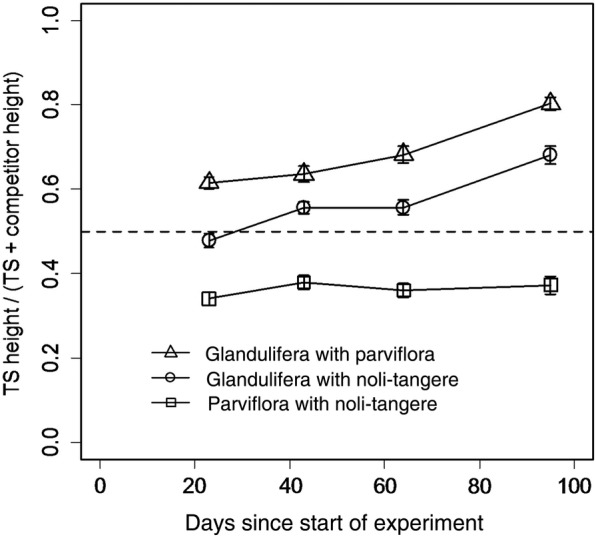
The temporal competition dynamics expressed as change in the ratio in target species height/(target plant + competitor height). TS, target species. At zero time the first seedlings emerged; height was first measured 22 days later. Triangle: *I. glandulifera* (target) with *I. parviflora* (competitor), circle: *I. glandulifera* with *I. noli-tangere*, square: *I. parviflora* with *I. noli-tangere*. The dashed line represents equal height of both competitors.

The height of the target plant, as well as the height ratio, expressed as the mean height of the target plant divided by mean height of the competitor + mean height of the target plant, was strongly influenced by competition in *I. noli-tangere* and *I. parviflora* during the experiment (Table 3) **[see Supporting Information—Table S4]**. *Impatiens glandulifera* overtopped both congeners from the early stages of the experiment and this difference became more pronounced with time. On the contrary, competition from both congeners did not affect the height of *I. glandulifera.* The plants of native *I. noli-tangere* competing with *I. parviflora* were taller throughout the experiment and the height ratio did not change markedly (Fig. 4).

## Discussion

### Performance as a function of competition, density and environment

Our results indicate that environmental variables and competition play a different role in the plant life-cycle completion and growth response of the three *Impatiens* species. Overall, competition was a more important factor than environmental conditions for all variables except for the life-cycle completion of plants over the growing season, which points to the importance of competitive interactions in evaluation of plant fitness and potential invasion success. This suggests that for the studied *Impatiens* species, the environment plays a role in early stages of the invasion process while competition becomes more important when it comes to the naturalization phase (Blackburn *et al*. 2011) and could act as a mechanism preventing the non-native species from colonizing the resident communities (Levine *et al*. 2004; Davies *et al*. 2010).

### Life-cycle completion

Life-cycle completion was surprisingly little affected by total plant density (with the only exception being a suppression of *I. parviflora* at high densities), indicating rather negligible self-thinning in our experimental populations. This contradicts reports from field studies, where a strong thinning to adult plant densities of *I. glandulifera* between 25 and 30 individuals/m^2^ from a seed rain of ∼5000–6000 seeds/m^2^ was observed (Perrins *et al*. 1990). The stronger thinning in the field can be attributed to seed predation and impact of other enemies (Dostál 2010), disturbances and large spatio-temporal heterogeneity in environmental factors, especially in soil moisture—factors from which plants are protected in an experimental garden. There is also a difference in the spatial pattern of seedling emergence; as seed dispersal in the field is random, seeds may emerge in dense patches, where it is impossible for the majority of plants to survive until maturity.

In contrast, environment had strong effect on life-cycle completion. *Impatiens glandulifera* performed poorly in moderate shade, if competing with the other invasive congener, *I. parviflora*; this could result from intensified light stress in the seedling stage due to a lack of shading by the low-statured seedlings of *I. parviflora*. Such a conclusion is supported by the fact that in the field *I. glandulifera* avoids full sunlight (Čuda *et al*. 2014). The native species *I. noli-tangere* generally showed the best performance of all three congeners in terms of the proportion of individuals that completed the life-cycle, which indicates that it may be better adapted to local conditions than the two alien species (Alexander *et al*. 2011). The number of individuals of *I. noli-tangere* that completed the life-cycle increased under high water supply (Čuda *et al*. 2014).

### Biomass and fecundity

In contrast to life-cycle completion, biomass and fecundity were strongly influenced by competition and slightly by environment. As expected, density had a strong negative effect on the biomass and fecundity of all species, which is in accordance with the law of constant final yield (Harper 1977), except for the fecundity of *I. glandulifera.* Although *I. glandulifera* was the poorest among all the species studied in terms of the life-cycle completion, the surviving plants were able to dominate the pots regardless of the competitor presence and abundance. The fecundity of *I. glandulifera* plants was not significantly affected by the congeners, both native and invasive, which also indicates this species' competitive superiority. The biomass of *I. glandulifera* decreased in competition with the native *I. noli-tangere*, but its fecundity remained unaffected despite a close correlation of the number of capsules with biomass. This contradiction can be interpreted as a sign of plasticity in allocation of assimilates into the seed production (Berg and Ellers 2010). Due to the limited occurrence of *I. glandulifera* in woodlands (Beerling and Perrins 1993), reflecting a higher demand for light than is available under dense canopies (Maule *et al*. 2000; Čuda *et al*. 2014), we expected lower fecundity of plants exposed to shade. However, the seed production was similar in both shading treatments. The ability of *I. glandulifera* to produce seeds until the very end of the growing season contributes to its superiority over its congeners. The biomass of *I. glandulifera* was not negatively influenced by low water supply (similar to Maule *et al*. 2000; Skálová *et al*. 2013; Čuda *et al*. 2014), despite this species being traditionally considered a water-demanding plant (Beerling and Perrins 1993). On the other hand, the biomass of *I. glandulifera* decreased in the high water treatment, a phenomenon possibly associated with the high water content in its stems. Water is important to maintain turgor in the supporting structures. High water content, ∼96 %, is maintained by nitrate accumulation, which is used as an osmoticum in stems and leaves (Andrews *et al*. 2009). If water supply is insufficient, the plants have to invest more into cellulose in the stem structure. This opinion is supported by the plants reaching similar height in the low and high water treatments. High water content due to nitrate accumulation in place of organic molecules in stems enables the species to achieve substantial height at low irradiance (Andrews *et al*. 2009) or for instance to invest the assimilates into increased fecundity. The biomass and fecundity of *I. parviflora* were reduced in competition with both congeners, more so if the competition was intense; this shows that this is the weakest competitor of the three species. The native *I. noli-tangere* produced less biomass and fewer capsules when grown alone than in competition with *I. parviflora*. This means that *I. noli-tangere* suffers more from intraspecific competition than from interspecific competition with *I. parviflora* and, therefore, it has limited impact on *I. noli-tangere* under most conditions except for strong water limitation (Skálová *et al*. 2012). This is contrary to Tichý (1997) and Faliński (1998), who supposed that *I. parviflora* could influence *I. noli-tangere* by competition, but did not test this hypothesis experimentally. Biomass and fecundity of *I. noli-tangere* decreased across all environmental treatments in competition with *I. glandulifera* and increased with *I. parviflora* compared with monospecific control. This indicates an intermediate position of the native species in the competitive hierarchy within the members of the genus occurring in the studied region, and its ability to resist the competition by the less invasive alien congener. However, its ability to resist is context specific. For example the presence and timing of disturbances is very important, because the species differ in the time of setting seeds. In general, *I. parviflora* suppresses *I. noli-tangere* in very dry conditions (Skálová *et al*. 2012) and *I. glandulifera* outcompetes it wherever *I. noli-tangere* is able to survive.

### Temporal variation in competition due to the difference in species height

Although *I. glandulifera* was not the tallest at the beginning of the experiment, it overtopped both congeners rather early and its superiority increased during the growing season. The ability of *I. glandulifera* to grow through the whole vegetation period facilitates its competitive dominance and also increases its propagule pressure, because plants flower and fruit from July to the first frost (Beerling and Perrins 1993). On the other hand, the height ratio between *I. parviflora* and *I. noli-tangere* was relatively consistent, with *I. parviflora* being shorter all the time.

## Conclusions

The results suggest that the effect of competitor density on the performance of invasive *Impatiens* species exceeds that of environmental factors. Competitive interactions with co-occurring congeners may be thus a more important predictor of the invasion success of an invasive species and its population dynamics than its response to abiotic factors, and should be taken into account when evaluating their invasion potential.

The high invasiveness of *I. glandulifera* seems to result from its competitive dominance over the other congeners across varying environmental conditions of light and moisture. A main mechanism underlying this species' success is fast growth resulting in tall stature, which enables the plants to exploit available light and ability to still growing over the whole vegetation period. On the other hand, success of *I. parviflora* is definitely not caused by its competitive strength, but probably by its ability to avoid competition by tolerance of extreme conditions. Competitive exclusion of the native species *I. noli-tangere* is likely to occur from the stands with co-occurring *I. glandulifera*, but in mixed stands with the other invasive congener, *I. parviflora*, the impact on the native species will probably be limited.

## Sources of Funding

This study was funded by Praemium Academiae award from the Academy of Sciences of the Czech Republic to P.P., long-term research development project RVO 67985939 (Academy of Sciences of the Czech Republic) and institutional resources of the Ministry of Education, Youth, and Sports of the Czech Republic.

## Contributions by the Authors

All listed authors wrote the manuscript; J.Č., H.S. and P.P. designed the experiment; J.Č. and H.S. collected the data and Z.J. conducted the statistical analyses.

## Conflict of Interest Statement

None declared.

## Supporting Information

The following additional information is available in the online version of this article –

**Table S1.** Effects of experimental conditions on life-cycle completion (the proportion of survived individuals from seed to maturity).

**Table S2.** Effects of experimental conditions on the average biomass of the individual.

**Table S3.** Effects of experimental conditions on fecundity (the average number of capsules produced by an individual).

**Table S4.** Effects of experimental conditions on the temporal variation, i.e. changes in the height ratio of the target species to the competitor during the duration of the experiment.

Additional Information
