# Supplementary material for: Competition among native and invasive Impatiens species: the roles of environmental factors, population density and life stage
Source: AoB Plants. 2015 Apr 1;7:plv033. doi: 10.1093/aobpla/plv033 (PMC4417208; doi:10.1093/aobpla/plv033)
Supplement: Additional Information [file supp_7_plv033_index.html]

Competition among native and invasive Impatiens species: the roles of environmental factors, population density and life stage — Additional Information 

# Competition among native and invasive *Impatiens* species: the roles of environmental factors, population density and life stage

## Additional Information

Additional Information

**Files in this Data Supplement:**

- Supplementary Table 1 - doc file
- Supplementary Table 2 - doc file
- Supplementary Table 3 - doc file
- Supplementary Table 4 - doc file
