# Supplementary material for: Competition among native and invasive Impatiens species: the roles of environmental factors, population density and life stage
Source: AoB Plants. 2015 Apr 1;7:plv033. doi: 10.1093/aobpla/plv033 (PMC4417208; doi:10.1093/aobpla/plv033)
Supplement: Additional Information [file supp_plv033_plv033supp_table1.doc]

**Table 1.** Effects of experimental conditions on life-cycle completion, i.e. the proportion of survived individuals from seed to maturity. Linear model. Explanatory variables abbreviations: dens = density, env = environmental treatment, comp = competitor identity, comp # = competitor density. Effect shortcuts: N = *I. noli-tangere*, P = *I. parviflora*, G = *I. glandulifera*, hw = high water, lw = low water, ms = moderate shade, ds = deep shade, h = high, l = low, + = positive, - = negative effect. Effects of water availability, shading and competitor identity were tested by posterior comparisons. Explained variance (EV) displayed only for significant factors, variance explained by all model in bold.

|  | ***I. noli-tangere*** | | | | ***I. parviflora*** | | | | ***I. glandulifera*** | | | |
| --- | --- | --- | --- | --- | --- | --- | --- | --- | --- | --- | --- | --- |
|  | D.f. | Effect | P | EV (%) | D.f. | Effect | P | EV (%) | D.f. | Effect | P | EV (%) |
| **Life-cycle completion** | | | | **35.5** |  | | | **27.7** |  | | | **23.9** |
| dens | 1 |  | 0.109 |  | 1 | h- | **0.050** | 0.8 | 1 |  | 0.127 |  |
| env | 2 | hw+ | **< 0.001** | 15.8 | 2 | ds+ | **< 0.001** | 9.4 | 2 | lw- | **0.038** | 1.4 |
| comp | 2 |  | 0.163 |  | 2 | G-N+ | **< 0.001** | 5.1 | 2 | P- | **< 0.001** | 4.1 |
| comp # | 1 | h- | **0.006** | 1.3 | 1 | h+ | **< 0.001** | 2.2 | 1 |  | 0.678 |  |
| dens × env | 2 | h×hw+ | **< 0.001** | 8.7 | 2 |  | 0.415 |  | 2 |  | 0.208 |  |
| dens × comp | 2 |  | 0.506 |  | 2 |  | 0.574 |  | 2 | l×P- | **0.003** | 2.5 |
| env × comp | 4 | ds×P+ | **0.006** | 2.6 | 4 | ms×G- | **0.014** | 2.5 | 4 | ms×P- | **< 0.001** | 8.7 |
| dens × comp # | 1 |  | 0.602 |  | 1 |  | 0.446 |  | 1 |  | 0.251 |  |
| env × comp # | 2 | hw×h + | **0.002** | 2.3 | 2 | ms×l- | **< 0.001** | 2.9 | 2 |  | 0.334 |  |
| comp × comp # | 1 |  | 0.643 |  | 1 | G-N+ | **0.006** | 1.5 | 1 |  | 0.130 |  |
| dens × env: comp | 4 |  | **0.009** | 2.4 | 4 |  | 0.190 |  | 4 |  | **0.008** | 3.0 |
| dens × env × comp # | 2 |  | 0.498 |  | 2 |  | 0.236 |  | 2 |  | 0.747 |  |
| dens × comp × comp # | 1 |  | 0.474 |  | 1 |  | 0.270 |  | 1 |  | 0.056 |  |
| env × comp × comp # | 2 |  | 0.169 |  | 2 |  | 0.626 |  | 2 |  | 0.334 |  |
| dens × env × comp × comp # | 2 |  | 0.793 |  | 2 |  | 0.196 |  | 2 |  | 0.784 |  |
| residuals | 371 |  |  | 64.5 | 346 |  |  | 72.3 | 284 |  |  | 76.1 |
