# Supplementary material for: Competition among native and invasive Impatiens species: the roles of environmental factors, population density and life stage
Source: AoB Plants. 2015 Apr 1;7:plv033. doi: 10.1093/aobpla/plv033 (PMC4417208; doi:10.1093/aobpla/plv033)
Supplement: Additional Information [file supp_plv033_plv033supp_table2.doc]

**Table 2.** Effects of experimental conditions on the average biomass of the individual. See Table 1 for abbreviations.

|  | ***I. noli-tangere*** | | | | ***I. parviflora*** | | | | ***I. glandulifera*** | | | |
| --- | --- | --- | --- | --- | --- | --- | --- | --- | --- | --- | --- | --- |
|  | D.f. | Effect | P | EV (%) | D.f. | Effect | P | EV (%) | D.f. | Effect | P | EV (%) |
| **Biomass** | | | | **54.7** |  | | | **58.8** |  | | | **22.7** |
| dens | 1 | h- | **< 0.001** | 23.4 | 1 | h- | **< 0.001** | 17.8 | 1 | h- | **< 0.001** | 8.2 |
| env | 2 | ds- | **< 0.001** | 3.9 | 2 | hw+ | **0.009** | 1.2 | 2 | hw- | **0.019** | 2.2 |
| comp | 2 | G-P+ | **< 0.001** | 20.6 | 2 | N-G- | **< 0.001** | 12.1 | 2 | N- | **< 0.001** | 7.1 |
| comp # | 1 |  | 0.008 | 0.9 | 1 | h- | **< 0.001** | 18.5 | 1 |  | 0.618 |  |
| dens × env | 2 |  | 0.455 |  | 2 |  | 0.056 |  | 2 |  | 0.804 |  |
| dens × comp | 2 |  | 0.202 |  | 2 | h×P+ | **0.013** | 1.1 | 2 |  | 0.518 |  |
| env × comp | 4 |  | 0.440 |  | 4 |  | 0.594 |  | 4 |  | 0.313 |  |
| dens × comp # | 1 |  | 0.253 |  | 1 | h×l+ | **< 0.001** | 2.7 | 1 |  | 0.657 |  |
| env × comp # | 2 | lw×h- | **0.005** | 1.4 | 2 |  | 0.102 |  | 2 | ms×N- | **0.049** | 1.7 |
| comp × comp # | 1 | G-P+ | **0.001** | 1.3 | 1 | N-G- | **< 0.001** | 1.9 | 1 |  | 0.667 |  |
| dens × env: comp | 4 |  | 0.139 |  | 4 |  | 0.349 |  | 4 |  | 0.552 |  |
| dens × env × comp # | 2 |  | 0.335 |  | 2 |  | 0.411 |  | 2 |  | 0.780 |  |
| dens × comp × comp # | 1 |  | 0.422 |  | 1 |  | **0.007** | 0.9 | 1 |  | 0.455 |  |
| env × comp × comp # | 2 |  | 0.549 |  | 2 |  | 0.978 |  | 2 |  | 0.915 |  |
| dens × env × comp × comp # | 2 |  | 0.143 |  | 2 |  | 0.423 |  | 2 |  | 0.612 |  |
| residuals | 351 |  |  | 45.3 | 341 |  |  | 41.2 | 277 |  |  | 77.3 |
