# Supplementary material for: Competition among native and invasive Impatiens species: the roles of environmental factors, population density and life stage
Source: AoB Plants. 2015 Apr 1;7:plv033. doi: 10.1093/aobpla/plv033 (PMC4417208; doi:10.1093/aobpla/plv033)
Supplement: Additional Information [file supp_plv033_plv033supp_table3.doc]

**Table 3.** Effects of experimental conditions on the fecundity, i.e. the average number of capsules produced by an individual. See Table 1 for abbreviations.

|  | ***I. noli-tangere*** | | | | ***I. parviflora*** | | | | ***I. glandulifera*** | | | |
| --- | --- | --- | --- | --- | --- | --- | --- | --- | --- | --- | --- | --- |
|  | D.f. | Effect | P | EV (%) | D.f. | Effect | P | EV (%) | D.f. | Effect | P | EV (%) |
| **Fecundity** | | | | **55.0** |  | | | **50.2** |  | | | **14.9** |
| dens | 1 | h- | **< 0.001** | 31.7 | 1 | h- | **< 0.001** | 13.1 | 1 |  | 0.125 |  |
| shading | 1 | ms+ | **0.003** | 2.9 | 1 |  | 0.063 |  | 1 |  | 0.898 |  |
| comp | 2 | P+G- | **< 0.001** | 10.1 | 2 | N-G- | **< 0.001** | 14.6 | 2 |  | 0.172 |  |
| comp # | 1 | h- | **0.010** | 2.2 | 1 | h- | **< 0.001** | 11.7 | 1 |  | 0.558 |  |
| dens × shading | 1 |  | 0.862 |  | 1 |  | 0.232 |  | 1 |  | 0.699 |  |
| dens × comp | 2 |  | 0.193 |  | 2 |  | 0.103 |  | 2 |  | 0.146 |  |
| shading × comp | 2 |  | 0.586 |  | 2 |  | 0.356 |  | 2 |  | 0.702 |  |
| dens × comp # | 1 |  | 0.117 |  | 1 | h×h- | **0.001** | 4.0 | 1 |  | 0.978 |  |
| shading × comp # | 1 |  | 0.088 |  | 1 |  | 0.399 |  | 1 |  | 0.289 |  |
| comp × comp # | 1 |  | 0.188 |  | 1 |  | 0.346 |  | 1 |  | 0.713 |  |
| dens × shading: comp | 2 |  | 0.057 |  | 2 |  | 0.399 |  | 2 |  | 0.054 |  |
| dens × shading × comp # | 1 |  | 0.111 |  | 1 |  | 0.464 |  | 1 |  | 0.466 |  |
| dens × komp × comp # | 1 |  | 0.058 |  | 1 |  | 0.136 |  | 1 |  | 0.908 |  |
| shading × komp × comp # | 1 |  | 0.411 |  | 1 |  | 0.372 |  | 1 |  | 0.264 |  |
| dens × shading × comp × comp # | 1 |  | 0.493 |  | 1 |  | 0.885 |  | 1 |  | 0.854 |  |
| residuals | 139 |  |  | 45 | 139 |  |  | 49.8 | 115 |  |  | 85.1 |
