# Supplementary material for: Competition among native and invasive Impatiens species: the roles of environmental factors, population density and life stage
Source: AoB Plants. 2015 Apr 1;7:plv033. doi: 10.1093/aobpla/plv033 (PMC4417208; doi:10.1093/aobpla/plv033)
Supplement: Additional Information [file supp_plv033_plv033supp_table4.doc]

**Table 4.** Effects of experimental conditions on the temporal variation, i.e. changes in the height ratio of the target species to the competitor during the duration of the experiment. The height ration formula: target species height / (target species + competitor height). See Table 1 for abbreviations.

|  | ***I. noli-tangere*** | | | | ***I. parviflora*** | | | | ***I. glandulifera*** | | | |
| --- | --- | --- | --- | --- | --- | --- | --- | --- | --- | --- | --- | --- |
|  | D.f. | Effect | P | EV (%) | D.f. | Effect | P | EV (%) | D.f. | Effect | P | EV (%) |
| **Temporal variation** | | | | **58.7** |  | | | **49.4** |  | | | **70.7** |
| pot | 334 |  | **< 0.001** |  | 304 |  | **< 0.001** |  | 287 |  | **< 0.001** |  |
| time | 1 |  | 0.181 |  | 1 | **-** | **< 0.001** | 10.8 | 1 | + | **< 0.001** | 68.8 |
| time × dens | 1 | h- | **< 0.001** | 1.8 | 1 |  | 0.072 | 0.2 | 1 |  | 0.671 |  |
| time × comp | 1 | G- P+ | **< 0.001** | 52.8 | 1 | G-N- | **< 0.001** | 33.0 | 1 |  | 0.059 |  |
| time × comp # | 1 | h- | **< 0.001** | 1.0 | 1 | h**-** | **< 0.001** | 0.9 | 1 |  | 0.896 |  |
| time × env | 2 | hw+ | **0.028** | 0.3 | 2 | hw+ | **0.003** | 0.8 | 2 | ds+ | **0.019** | 0.3 |
| time × dens × comp | 1 | l×P+ | **< 0.001** | 0.8 | 1 | l×G- | **0.017** | 0.4 | 1 | l×P+ | **0.044** | 0.2 |
| time × dens × comp # | 1 |  | 0.727 |  | 1 | l×h- | **0.049** | 0.3 | 1 |  | 0.096 |  |
| time × comp × comp # | 1 | P-G- | **< 0.001** | 0.9 | 1 | G×h- | **0.001** | 0.8 | 1 |  | 0.733 |  |
| time × dens × env | 2 |  | 0.697 |  | 2 |  | 0.495 |  | 2 | l×hw+ | **0.039** | 0.3 |
| time × comp × env | 2 |  | 0.709 |  | 2 |  | 0.625 |  | 2 | P×ds- | **0.012** | 0.4 |
| time × comp # × env | 2 |  | 0.761 |  | 2 | h×hw- | **0.022** | 0.5 | 2 |  | 0.692 |  |
| time × dens × komp × comp # | 1 |  | 0.234 |  | 1 |  | 0.278 |  | 1 |  | 0.750 |  |
| time × dens × comp × env | 2 |  | **0.014** | 0.4 | 2 |  | 0.202 |  | 2 |  | 0.437 |  |
| time × dens × comp # × env | 2 |  | 0.484 |  | 2 |  | 0.213 |  | 2 |  | 0.119 |  |
| time × comp × comp # × env | 2 |  | 0.149 |  | 2 |  | **< 0.001** | 1.1 | 2 |  | 0.473 |  |
| time × dens × comp × comp # × env | 2 |  | 0.340 |  | 2 |  | 0.480 |  | 2 |  | 0.390 |  |
| residuals | 862 |  |  | 41.3 | 772 |  |  | 50.6 | 689 |  |  | 29.3 |
